# Over-18%-Efficiency Quasi-2D Ruddlesden–Popper Pb–Sn Mixed Perovskite Solar Cells by Compositional Engineering

**DOI:** 10.1021/acsenergylett.3c00853

**Published:** 2023-06-28

**Authors:** Zhaotong Qin, Mike Pols, Minchao Qin, Jianquan Zhang, He Yan, Shuxia Tao, Xinhui Lu

**Affiliations:** † Department of Physics, The Chinese University of Hong Kong, Shatin 999077, Hong Kong SAR, People’s Republic of China; ‡ Materials Simulation and Modelling, Department of Applied Physics, Eindhoven University of Technology, P.O. Box 513, 5600MB Eindhoven, The Netherlands; § Department of Chemistry, Hong Kong University of Science and Technology, Kowloon 999077, Hong Kong SAR, People’s Republic of China

## Abstract

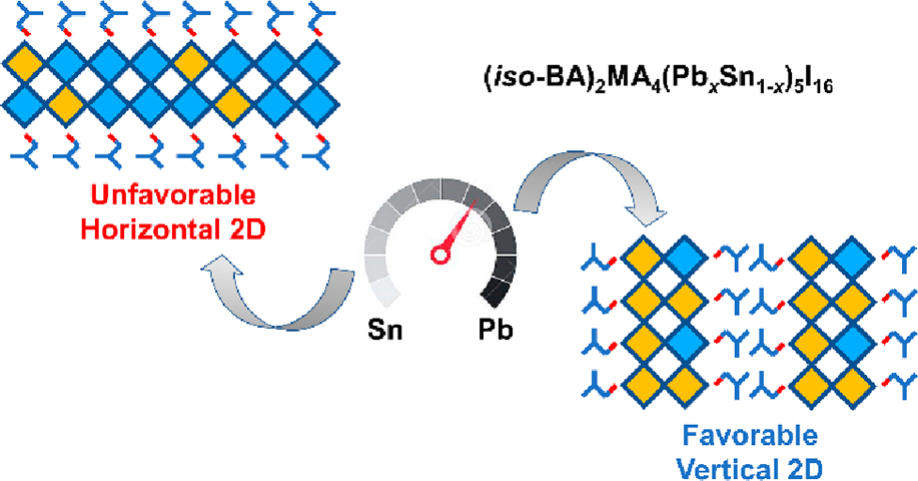

Quasi-two-dimensional (2D) Pb–Sn mixed perovskites show great potential in applications of single and tandem photovoltaic devices, but they suffer from low efficiencies due to the existence of horizontal 2D phases. Here, we obtain a record high efficiency of 18.06% based on 2D ⟨*n*⟩ = 5 Pb–S*n* mixed perovskites (*iso*-BA_2_MA_4_(Pb_*x*_Sn_1–*x*_)_5_I_16_, *x* = 0.7), by optimizing the crystal orientation through a regulation of the Pb/Sn ratio. We find that Sn-rich precursors give rise to a mixture of horizontal and vertical 2D phases. Interestingly, increasing the Pb content can not only entirely suppress the unwanted horizontal 2D phase in the film but also enhance the growth of vertical 2D phases, thus significantly improving the device performance and stability. It is suggested that an increase of the Pb content in the Pb–Sn mixed systems facilitates the incorporation of *iso*-butylammonium (*iso*-BA^+^) ligands in vertically oriented perovskites because of the reduced lattice strain and increased interaction between the organic ligands and inorganic framework. Our work sheds light on the optimal conditions for fabricating stable and efficient 2D Pb–Sn mixed perovskite solar cells.

Lead-tin (Pb–Sn) mixed halide perovskites hold great promise for both single-junction and tandem solar cells due to their tunable bandgaps in the ideal range of 1.1–1.4 eV.^1−4^ The power conversion efficiency (PCE) of three-dimensional (3D) Pb–Sn mixed perovskite solar cells (PSCs) has been pushed to as high as 23.6% through composition engineering,^5^ surface passivation,^6,7^ optimization of carrier transporting layer,^8^ etc. The regulation of the Pb/Sn ratio and the spatial distribution of Pb and Sn are crucial factors that affect the Pb–Sn perovskite film quality and resulting device performance. Snaith et al.^109^ reported a “defective zone” of the Sn concentration between 0.5%–20% where the optoelectronic properties deteriorate significantly. Furthermore, Yan et al. created a vertical gradient of the Pb/Sn ratios through a multitemperature antisolvent method, which improved the photocarrier extraction and thus PSC device efficiency.^9^ Despite the excellent optoelectronic properties of Pb–Sn mixed perovskite films, the incorporation of tin inevitably causes a drastic decrease of device stability due to the facile oxidation of Sn(II) to Sn(IV), which is further exacerbated by moisture.^10^

To address this issue, the deposition of highly ordered two-dimensional (2D) perovskites is an effective approach to protect perovskites from moisture and retard Sn(II) oxidation with the aid of hydrophobic ligands.^11−15^ Nonetheless, the insulating nature of long-chain ammonium ligands may impede the charge carrier transport in 2D perovskites, and thus the growth of a vertically oriented 2D crystal is critical.^13,16^ For Pb-based 2D perovskites, numerous strategies have been developed to regulate crystal orientation including additive incorporation,^17−20^ solvent engineering,^21,22^ modulation of ligand structures,^23,24^ etc. However, explorations of Pb–Sn mixed 2D perovskites are very limited, and the reported efficiencies are still below 10%. Chen et al. investigated film morphology, orientation, and charge transfer in (BA)_2_(MA)_3_Pb_4–*x*_Sn_*x*_I_13_ 2D perovskites with different Pb/Sn ratios, with a peak efficiency of 5.96%.^25^ To obtain high-efficiency and stable 2D Pb–Sn mixed PSCs, a rational selection of organic ligands^26^ and the precise control of the Pb/Sn ratio is crucial for regulating the crystal orientation in 2D Pb–Sn mixed perovskites, yet studies on these aspects are still missing.

In this work, we fabricate 2D Ruddlesden–Popper (RP) Pb–Sn mixed perovskites (⟨*n*⟩ = 5) with desired vertical orientation by utilizing *iso*-butylammonium (*iso*-BA^+^) ligands and tuning the Pb/Sn ratio, achieving a record-high efficiency of 18.07% for 2D Pb–Sn mixed PSCs. We show that the Pb/Sn ratio plays an important role in manipulating the crystal growth orientation, optoelectronic properties, and film morphology of 2D Pb–Sn mixed perovskites. The incident-angle dependent grazing-incidence wide-angle X-ray scattering (GIWAXS) measurements allow the investigation of the depth profiles in 2D perovskite films with different Pb/Sn ratios. We find that the Sn-rich films consist of a capping layer of 3D perovskites and a bottom layer of mixed horizontal- and vertical-oriented 2D perovskites. In contrast, the unwanted horizontal 2D perovskites can be suppressed in Pb-rich films. Theoretical calculations explain these results through increased ionic interactions between the organic cations and inorganic framework and smaller levels of strain required for the vertical growth of the 2D perovskite in Pb-rich films. Time-of-flight secondary ion mass spectroscopy (ToF-SIMS) results further disclosed the vertical variation in Pb–Sn ratio and ⟨*n*⟩ distribution. We highlight that the *iso*-BA^+^ based 2D Pb–Sn perovskites demonstrate stability superior to that of the 3D counterparts under high humidity and light illumination.

We choose ⟨*n*⟩ = 5 as the nominal *n* of the 2D perovskite due to its highest performance of the Pb-based PSCs as the benchmark (Figure S1). We first fabricated a series of 2D RP perovskites with the formula of *iso*-BA_2_MA_4_(Pb_*x*_Sn_1–*x*_)_5_I_16_, where *x* equals 0%, 10%, 30%, 50%, 70%, or 100%, by the hot-casting method.^13^ For simplicity, we abbreviate them as Pb0, Pb10, Pb30, Pb50, Pb70, and Pb100, respectively. GIWAXS measurements were conducted to investigate the influence of the Pb/Sn ratio on the crystal growth of the 2D Pb–Sn mixed perovskite films, as presented in Figure 1a. Detailed illustrations of different types of intensity profiles are shown in Figure S2. It is found that Sn-rich films (Pb0 and Pb10) show randomly oriented crystals, as demonstrated by the isotropic scattering rings in the GIWAXS patterns. In contrast, typical discrete scattering spots are observed when the Pb content reaches or exceeds 30% (Pb30–Pb100), indicative of the formation of highly oriented crystals,^13^ as illustrated in Figure 1b. To quantify the orientational order of the crystals (white arrows in Figure 1a), we extracted polar intensity profiles (Figure S3a) from the GIWAXS patterns for the (101) perovskite peak located at around 1.01 Å^–1^ and calculated the corresponding Hermans’ orientation factor (H-factor) in Figure S3b. The Sn-rich Pb0 and Pb10 films exhibit low H-factors of 0.048 and 0.042, respectively, suggesting a random orientation of corner-sharing [MI_6_] octahedra (M = Pb_*x*_Sn_1–*x*_).^27−29^ The H-factor is greatly enhanced when the concentration of Pb reaches 30% (Pb30) and keeps increasing from 0.56 (Pb30) to 0.81 (Pb100), indicating a higher degree of orientational order as the lead content rises. In short, the elevated lead content facilitates the growth of highly ordered 2D *iso*-BA_2_MA_4_(Pb_*x*_Sn_1–*x*_)_5_I_16_ perovskite crystals. Meanwhile, the Pb-rich films (i.e., Pb70 and Pb100) demonstrate enhanced film crystallinity, as indicated by the larger (101) peak areas (Figure 1d).

**Figure 1:**
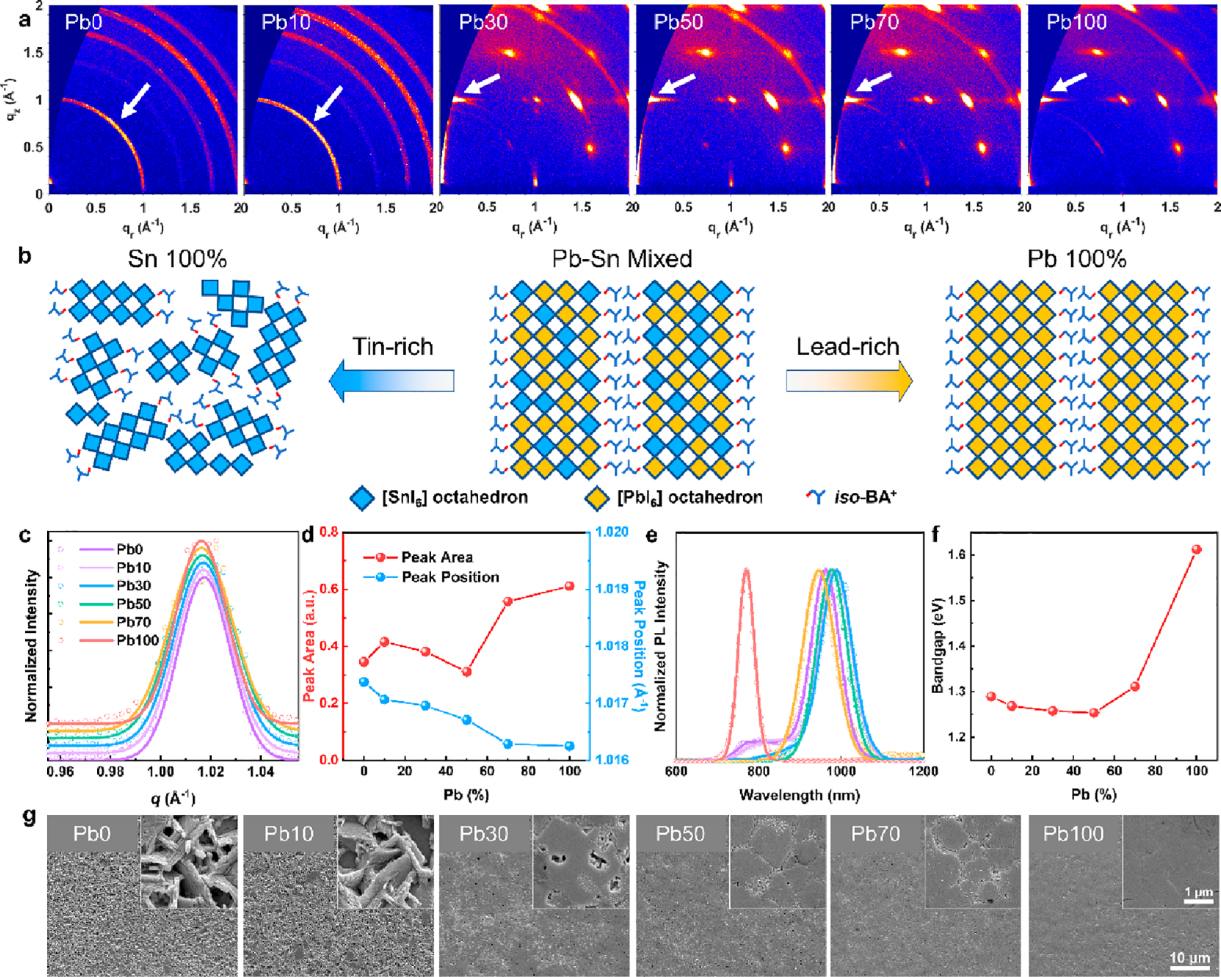
(a) GIWAXS patterns of 2D *iso*-BA_2_MA_4_(Pb_*x*_Sn_1–*x*_)_5_I_16_ (x = 0%, 10%, 30%, 50%, 70%, or 100%) perovskite films. The white arrows indicate the (101) plane of the 2D perovskites. (b) Schematic illustrations of influences of the Pb/Sn ratio on the crystal structure of 2D Pb–Sn mixed perovskites. (c) The corresponding intensity profiles of (a) enlarged in the *q* range of 0.95–1.06 Å^–1^. (d) Changes of perovskite peak areas and peak positions along with different Pb contents. (e) Normalized photoluminescence (PL) spectra and (f) the corresponding estimated bandgaps of the 2D *iso*-BA_2_MA_4_(Pb_*x*_Sn_1–*x*_)_5_I_16_ films. (g) Surface SEM images of the 2D *iso*-BA_2_MA_4_(Pb_*x*_Sn_1–*x*_)_5_I_16_ perovskite films.

As the lead content increases, we also observed that the perovskite peak shifts toward lower diffraction angles in Figure 1c,d, as the incorporation of larger Pb ions leads to expansion of the Pb–Sn mixed perovskite crystal structure. The changes of the crystal structure and composition affect the optoelectronic properties of the films.^30^ Therefore, photoluminescence (PL) spectra were measured (Figure 1e) to study the change of bandgaps with different Pb/Sn ratios. As summarized in Figure 1f, a nonlinear change in bandgap, the so-called “bowing effect” in 3D Pb–Sn mixed perovskites,^4^ is also observed in these 2D Pb–Sn mixed perovskites. The smallest bandgap of 1.25 eV is achieved by the Pb50 film rather than the pure Sn-based film (Pb0, 1.29 eV), owing to the difference in energy level offsets of the s and p orbitals of Sn and Pb that form the band edges of the alloy.^31−33^ Except for the Pb100 film with a particularly large bandgap of 1.61 eV, all the other tin-containing samples present low bandgaps of around 1.30 eV, which are within the optimal bandgap range for solar cells.^1,2^ It is noted that the bandgaps of the 2D *iso*-BA_2_MA_4_(Pb_*x*_Sn_1–*x*_)_5_I_16_ perovskite films are slightly larger than their 3D counterparts due to the quantum confinement effect.^12^ For the Pb100 2D perovskite, we observed several excitonic peaks originating from different ⟨*n*⟩ members in the absorption and photoluminescence spectra (Figure S4). Nonetheless, the lead–tin mixed 2D perovskites (i.e., Pb70 perovskites) present negligible excitonic absorption and emission in absorption and PL spectra, which may be due to the lower exciton binding energy in lead–tin mixed quasi-2D perovskites.^34^

The influence of the Pb:Sn ratio on the 2D Pb–Sn mixed perovskite film morphology was further investigated by scanning electron microscopy (SEM), as shown in Figure 1g. The tin-rich Pb0 and Pb10 films show rough film surfaces with poor coverage, presumably due to the fast uncontrollable crystallization kinetics of tin-rich perovskites.^35−37^ The discontinuous and unoriented slablike crystals in Pb0 and Pb10 films can be observed more clearly in Figure S5. The film coverage is greatly improved in the Pb30 film, while there are still some pinholes that do not disappear until the Pb content is further increased to 50% (Figure 1g). The perovskite films with higher Pb contents (i.e., Pb70 and Pb100) exhibit smooth and compact surface morphologies. Nonetheless, buried voids within the perovskite films are still observed in cross-sectional SEM images in Figure 2a,b (marked by white arrows), while the volume of the buried voids is gradually reduced from the Pb30 to Pb100 films.

**Figure 2:**
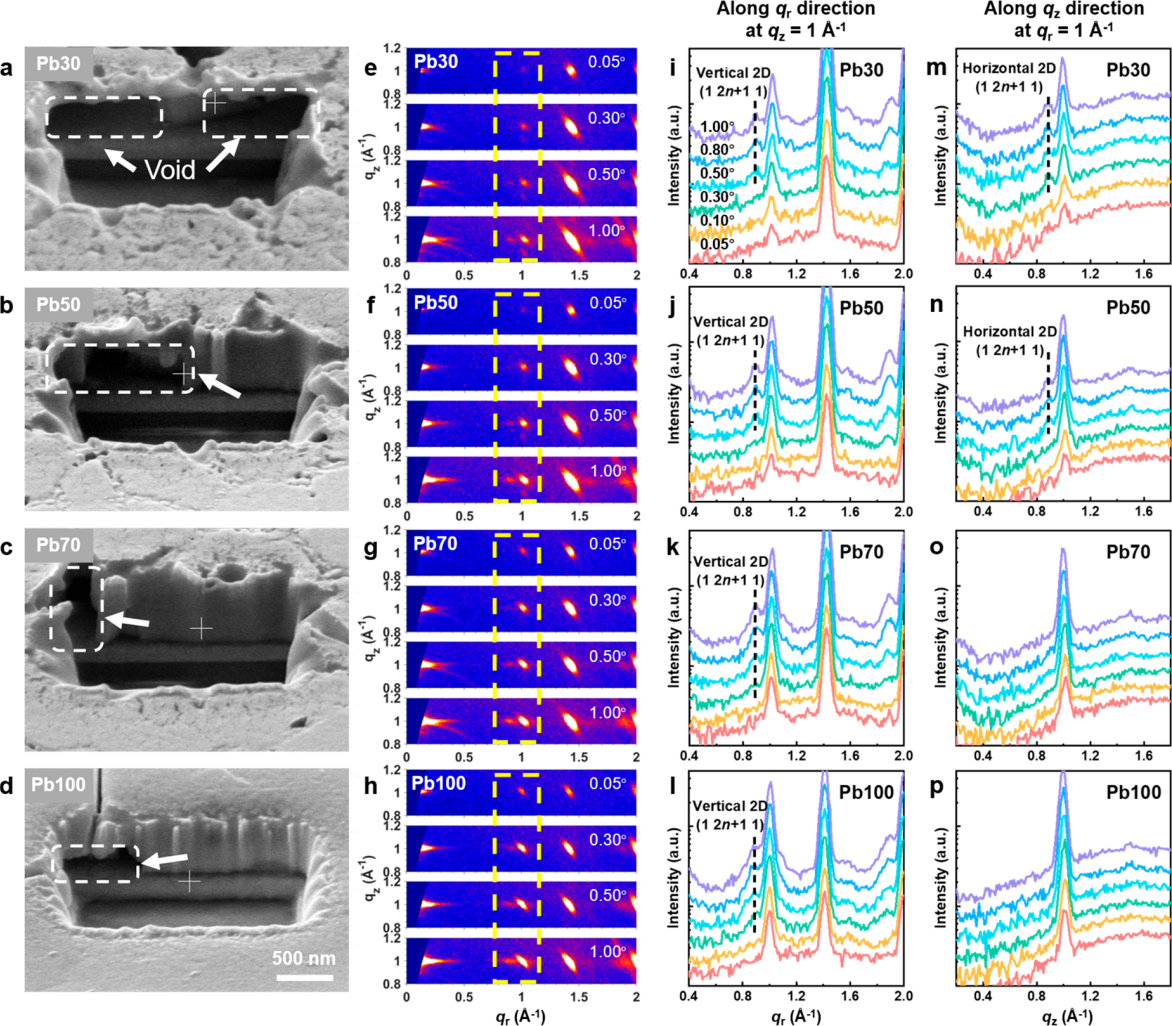
(a–d) Cross-sectional SEM images of the focused ion beam (FIB) prepared 2D (a) Pb30, (b) Pb50, (c) Pb70, and (d) Pb100 perovskite films. (e–h) GIWAXS patterns in the *q*_*z*_ range 0.8–1.2 Å^–1^ of the 2D (e) Pb30, (f) Pb50, (g) Pb70, and (h) Pb100 perovskite films measured under different incident angles and the corresponding GIWAXS intensity profiles (i–l) along the *q*_r_ direction at *q*_*z*_ = 1 Å^–1^ and (m–p) along the *q*_*z*_ direction at *q*_r_ = 1 Å^–1^.

In order to study the phase distribution along the thickness direction under different Pb/Sn ratios, we performed GIWAXS depth profiling measurements by adjusting the X-ray penetration depth by varying the incident angle from 0.05° to 1.0° (Figure S6).^38^Figure 2e–h presents enlarged GIWAXS patterns in the *q*_*z*_ range of 0.8–1.2 Å^–1^ in order to focus on the specific signals from the vertical and horizontal 2D phases^26^ at different depths of the films, and the full range of the GIWAXS patterns are shown in Figure S7. The corresponding intensity profiles along *q*_r_ at *q*_*z*_ = 1 Å^–1^, along *q*_*z*_ at *q*_r_ = 1 Å^–1^, and along the polar angle χ at *q* = 1 Å^–1^ are displayed in Figure 2i–l, Figure 2m–p, and Figure S8, respectively. Detailed illustrations of different types of intensity profiles are shown in Figure S2. It is noted that due to the discontinuous surface with poor morphology, the incident X-ray could penetrate the whole thickness of the Sn-rich Pb0 and Pb10 films at any incident angle providing no depth sensitivity. As for the Pb30 film, GIWAXS patterns obtained with shallow incident angles of 0.05° and 0.10° only show scattering peaks from the 3D phase^26^ (Figure 2e,i,m), indicating that the surface of the 2D Pb–Sn mixed film is predominantly 3D phase-enriched, which is consistent with previous reports.^21,39^ The same phenomenon is also observed in the other three 2D perovskite films (i.e., Pb50, Pb70, and Pb100). As the incident angle increases to 0.3°, the X-ray penetration depth goes deeper to around 200 nm, where the characteristic (1,2*n*+1,1) peak (see Figure S9 for the labeling) of the horizontal 2D perovskite phase appears first in the Pb30 film (Figure 2m). Subsequently, the peak of the vertical 2D perovskite phase emerges at a higher incident angle of 0.5°, with a penetration depth of around 300 nm (Figure 2i). Further increasing incident angles to 0.8° and 1° enables full penetration of the X-rays into the film, as demonstrated by the detected ITO peak at *q* ≈ 2.1 Å^–1^ (Figure S7). Signals from both horizontal and vertical 2D perovskite phases coexist in the GIWAXS patterns measured under large incident angles of 0.8° and 1°. The GIWAXS profiling results confirm that the Pb30 film possesses a capping layer of the 3D perovskites and a bottom layer of horizontal- and vertical-oriented 2D perovskites. It is worth mentioning that the horizontal 2D perovskite phase whose ligands block the transport of photogenerated carriers is not favored in photovoltaic devices.^16^

When the Pb content increases to 50% (Pb50), the distribution of the horizontal 2D perovskite phase along the thickness direction is slightly restrained considering that the corresponding (1,2*n*+1,1) peak appears at a larger incident angle of 0.5° (Figure 2n) compared with the Pb30 film. Promisingly, the horizontal 2D phase is completely suppressed in the Pb-rich Pb70 and Pb100 films (Figure 2o,p). In the meantime, the peak of the vertical 2D phase is observed at a smaller incident angle of 0.3° (Figure 2k,l) and intensified with the increase in the penetration depth. This indicates that the raised Pb content in 2D Pb–Sn mixed perovskites can not only suppress the formation of the unwanted horizontal 2D perovskite phase but also promote the growth of the desired vertical 2D perovskite phase at the bottom of the perovskite film. It is noted that the surface of the Pb70 and Pb100 films is dominated by 3D perovskites as well in light of the GIWAXS data measured under shallow incident angles. Therefore, the Pb-rich Pb70 and Pb100 films also exhibit a layered structure that is composed of a similar capping layer of 3D perovskites and a bottom layer of only vertically oriented 2D perovskites.

ToF-SIMS measurements (Figure S10) were further conducted to confirm the 3D/2D phase distribution in the 2D Pb–Sn mixed perovskite films. We calculated the intensity ratios of MA/BA to reveal the relative ⟨*n*⟩ member distribution, as shown in Figure 3a–d. Note that the MA/BA ratio does not directly correspond to the absolute ⟨*n*⟩ value, but only the relative changing trend of perovskite ⟨*n*⟩ phases.^40^ Generally, a higher MA/BA ratio represents a larger average ⟨*n*⟩ value of perovskites. The 3D perovskite phase can be regarded as larger-⟨*n*⟩ perovskite species (⟨*n*⟩ is close to infinite), while the 2D perovskites can be regarded as smaller-⟨*n*⟩ perovskite species (typically ⟨*n*⟩ ≤ 5). It is found that, for all four 2D Pb–Sn mixed perovskite films, the MA/BA ratio is much high near the film surface (Figure 3a–d), implying that larger ⟨*n*⟩ perovskite phases are enriched at the surface,^41,42^ consistent with the observed dominant 3D phase signals. Furthermore, the MA/BA ratio decreases to a low level as the sputtering time increases to a certain point, consistently manifesting the enrichment of low ⟨*n*⟩ phases (i.e., low-dimensional 2D perovskites) at the bottom of the film.^41,42^ The ToF-SIMS results further confirm the layered 3*D*/2D structure in 2D Pb–Sn mixed perovskite films.

**Figure 3:**
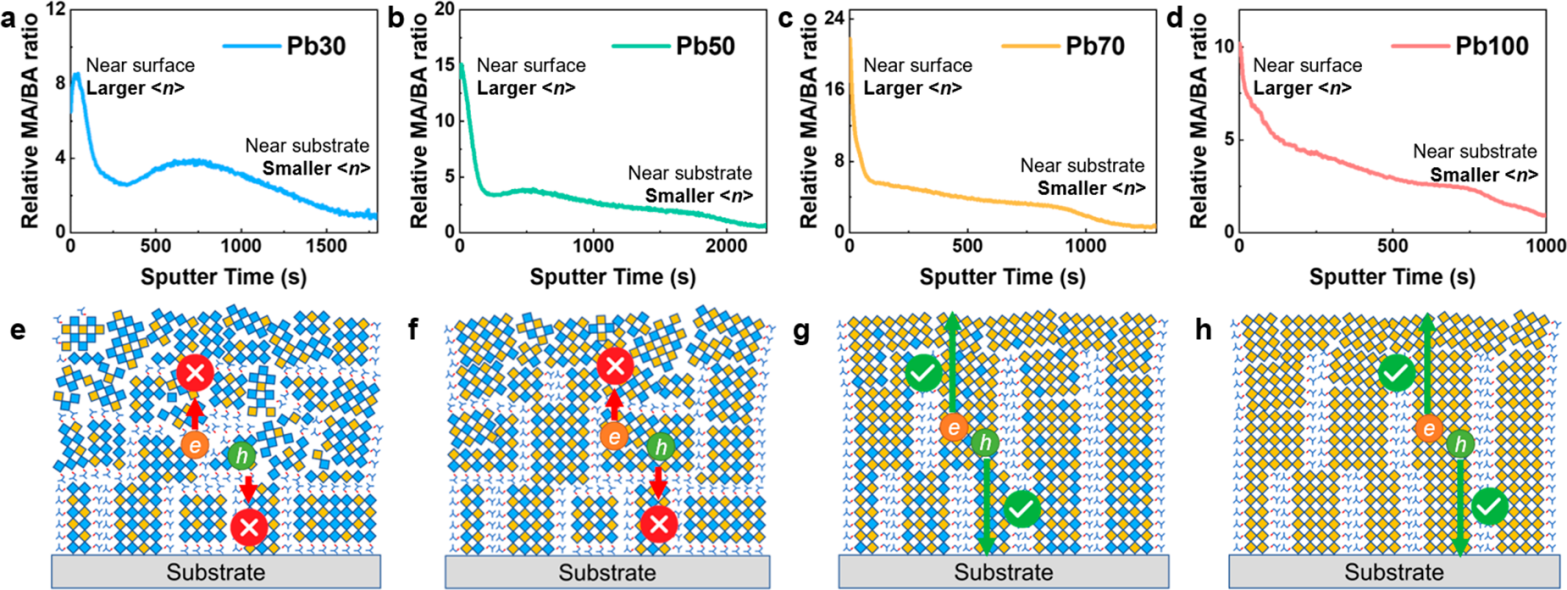
(a–d) Plots of the relative MA/BA ratio versus the sputtering time of different 2D *iso*-BA_2_MA_4_(Pb_*x*_Sn_1–*x*_)_5_I_16_ perovskite films based on ToF-SIMS measurements. (e–h) Schematics of the distributions of 3D and horizontal/vertical 2D phases along the thickness direction in (e) Pb30, (f) Pb50, (g) Pb70, and (h) Pb100 films, in which yellow rhombi represent [PbI_6_] octahedra, blue rhombi represent [SnI_6_] octahedra, branched patterns represent *iso*-BA^+^ ligands, and MA^+^ ions are not shown.

We also found that the layered film structure affects the orientational order of [MI_6_] stacking along the thickness direction, as revealed by the changing H-factors of the (101) plane along different film depths (Figure S11). H-factors of all four films gradually increase from surface to bottom, indicative of a vertically enhanced crystal orientational order. This corresponds to the layered film structure, in which the larger-⟨*n*⟩ (e.g., 3D) perovskites distributed on the film surface lead to a less oriented [MI_6_] stacking while the smaller-⟨*n*⟩ perovskites at the bottom of the film give rise to improved crystal orientation, which is consistent with our findings. The overall orientational order of [MI_6_] stacking increases as the lead content increases, which is consistent with the conclusion in Figure S3. Remarkably, the Pb-rich films (i.e., Pb70 and Pb100) possess similarly high H-factors among the four samples throughout the whole thickness range, demonstrating the highest orientational order of [MI_6_] stacking.

Combining GIWAXS and ToF-SIMS results, the vertical phase distribution and crystal orientation in different 2D *iso*-BA_2_MA_4_(Pb_*x*_Sn_1–*x*_)_5_I_16_ perovskite films are illustrated in Figure 3e–h. All films exhibit the 3D perovskite phase on the top surface and 2D perovskite phases on the bottom of the film. The Pb30 and Pb50 2D films show a poor crystal orientation order throughout the whole film. There exist horizontal 2D perovskite phases in both Pb30 and Pb50 films, which obstruct the charge transport in perovskite films due to the horizontally aligned insulating ligands. In contrast, the Pb-rich films (i.e., Pb70 and Pb100) have better orientational order and are free of unwanted horizontal 2D phases are absent. Instead, vertical 2D perovskite phases are predominant at the bottom, contributing to efficient charge transport and potentially improved device performance.

Besides, DFT calculations (see Supporting Notes, Figure S12, and Tables S1–S4 in the Supporting Information) were performed to further explain the possible reason for the orientational tendency of 2D perovskite formation. The orientational contrast can be explained by the different chemical bonding nature in Pb- and Sn-based perovskites. Sn has stronger covalent interactions with I than Pb does, thus promoting smaller nucleation centers and faster growth. Sn is also smaller than Pb in size. Consequently, large ligands fit more poorly in the SnI_*x*_ inorganic framework, inducing a lattice strain. This explains Sn-rich precursors tend to form nanosized disordered phases without any long-range vertical growth. When increasing Pb content, the ionic interactions become more dominant and ligand–framework interactions become stronger. As a result, the growth in the vertical direction is promoted, which is further aided by reduced lattice strain because of slightly larger size of Pb.

We fabricated PSCs based on 2D Pb–Sn mixed perovskite films from Pb0 to Pb100 with an inverted structure of ITO/PEDOT:PSS/Perovskite/PC_61_BM/BCP/Ag to evaluate the device performance. The devices based on Pb0 and Pb10 films show extremely low open-circuit voltage (*V*_oc_) and short-circuit current density (*J*_sc_) due to the poor film morphology (Figure 1g and Figure S5), resulting in PCEs of almost 0% (Figure S13). The Pb30 and Pb50 based PSCs also suffer from low efficiencies (Figure 4a) which can be ascribed to relatively poor crystal orientation and the existence of horizontal 2D perovskite phases. Specifically, the low orientational order in Pb30 and Pb50 films may increase the trap-assisted recombination loss in the devices according to the high ideality factors^43−46^ in the light-intensity dependence *V*_OC_ measurements in Figure 4b. Meanwhile, the horizontal 2D perovskites in Pb30 and Pb50 films impede the charge transport in the devices, as revealed by the larger charge transport/transfer resistance (*R*_ct_) estimated from the electrochemical impedance spectroscopy (EIS) results in Figure 4c. In contrast, the Pb-rich PSCs (Pb70 and Pb100) exhibit much higher PCEs with closer-to-1 ideality factors (Figure 4b) and smaller *R*_ct_ values (Figure 4c, Figure S14 and Table S5), implying the suppressed trap-assisted recombination and efficient charge extraction in Pb70 and Pb100 films. This can be attributed to the improved film quality in terms of better film crystallinity, enhanced crystal orientation, and predominant vertical-2D perovskites. Notably, the bandgap of the Pb70 film (1.31 eV) is much narrower than that of the Pb100 film (1.61 eV), giving rise to higher *J*_SC_ (Figure S15 and Table S6) but lower *V*_OC_ (Figure S13). As a consequence, the Pb70 based PSCs demonstrate the highest efficiency of around 16%. Remarkably, the Pb70 devices exhibit the least *V*_oc_ loss of 0.497 V (Table S7), which is further reduced to 0.427 V by optimizing the electron transporting materials (PC_61_BM:PC_71_BM:ICBA = 1:1:12, w/w/w), resulting in a record high efficiency of 18.07% (Figure 4d, Figures S16 and S17, and Table S8), which is the highest PCE of 2D lead–tin mixed PSCs reported so far.

**Figure 4:**
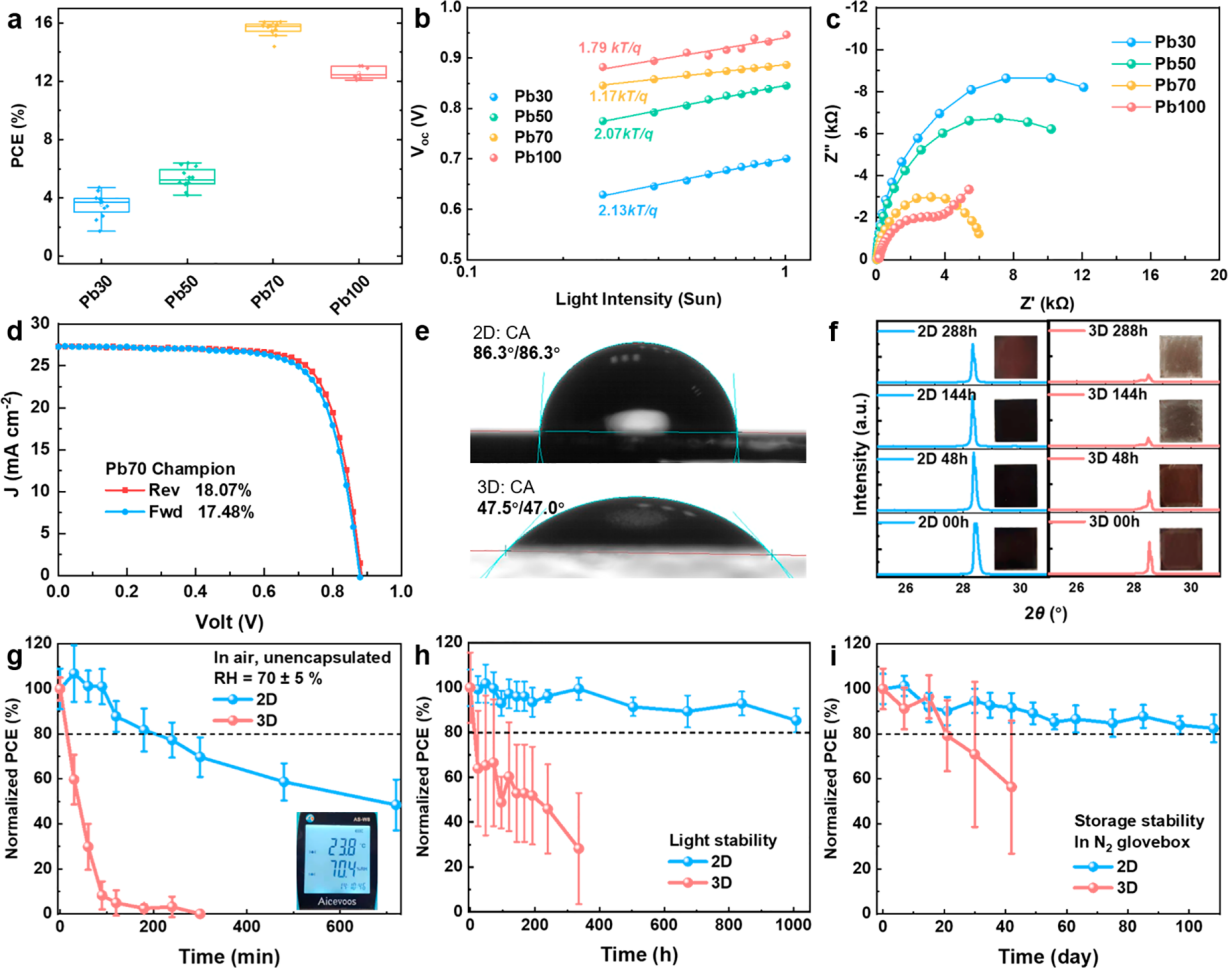
(a) Statistical PCE of PSCs based on the 2D Pb–Sn mixed perovskites from Pb30 to Pb100. (b) Plots of light-intensity dependent *V*_oc_. (c) EIS curves measured under light illumination and 0 V bias. (d) *J–V* curves of a champion device (Pb70) measured under forward and reverse scans. (e) Contact angle measurements performed on 2D and 3D Pb–Sn mixed perovskite films with a Pb content of 70% (Pb70). (f) XRD spectra and the corresponding film photos of the 2D and 3D Pb70 perovskite films during the aging test in a high-humidity environment (RH = 70 ± 5%). (g–i) Stability tests of the unencapsulated 2D and 3D Pb70 PSCs (g) stored at RH = 70 ± 5% in air, (h) under 0.5 sun light illumination in a nitrogen-filled glovebox, and (i) under dark in a nitrogen-filled glovebox.

In order to demonstrate the superiority of 2D Pb–Sn mixed perovskites (Pb70) over their 3D counterparts in terms of film and device stability, we fabricated 3D Pb–Sn mixed MA(Pb_0.7_Sn_0.3_)I_3_ perovskite films with the same Pb content of 70%. It was found that the hydrophobic ammonium *iso*-BA^+^ ligands in 2D Pb70 perovskites can prevent the penetration of moisture into the films, as proven by the increased water contact angle (Figure 4e). It is noted that although the film shows a 3D perovskite-rich surface, there still exist hydrophobic ligands at the film surface, as manifested by the ToF-SIMS results in Figure S10, thus leading to an increased water contact angle. Therefore, the 2D Pb70 film experienced a much smaller intensity drop for the perovskite peak at ∼28.3° in XRD spectra (Figure 4f) during the accelerated aging process in damp air with RH of 70 ± 5%, implying the excellent humidity stability of the 2D Pb70 film. By contrast, the perovskite peak of the 3D MA(Pb_0.7_Sn_0.3_)I_3_ film quickly diminished, and the black film faded after 288 h of storage in damp air. Accordingly, unencapsulated PSCs based on 2D Pb70 perovskite films retained over 80% of the initial efficiency after 200 min in damp air (∼70% RH) (Figure 4g) whereas the 3D counterparts lost over 90% in efficiency within 120 min. Besides, the 2D Pb70 based PSCs demonstrate device stability superior to that of the 3D-based PSCs under light illumination (Figure 4h) and the dark condition (Figure 4i), as well as at maximum power point (MPP) (Figure S18). The enhanced light stability is possibly due to the lifted activation energy of light-induced ion migration by ligand layers.^47,48^

In summary, we demonstrate a high efficiency of 2D Pb–Sn mixed PSCs by fabricating high-quality 2D perovskite films with a vertical crystal orientation. With the aid of SEM, GIWAXS and ToF-SIMS, we show that by tuning the Pb/Sn ratio in the 2D *iso*-BA_2_MA_4_(Pb_*x*_Sn_1–*x*_)_5_I_16_ mixed perovskites, film properties in terms of crystal orientation, optoelectronic properties, and film morphology can be modulated. For Sn-rich perovskites, mixed horizontal and vertical 2D perovskite phases form. In contrast, by increasing the Pb content up to 70%, the growth distinctively favors a vertical growth direction and eventually forms films with desired crystal orientation and improved film crystallinity. Out of all investigated compositions, Pb70 mixed perovskite films exhibit close-to-ideal bandgaps, the lowest trap densities, and lowest charge extraction resistances, resulting in PCSs with a champion PCE of 18.06%, by far the highest PCE of 2D Pb–Sn mixed PSCs. Compared with the 3D Pb–Sn counterpart, the 2D Pb–Sn mixed perovskites also demonstrate superior shelf stability under high humidity (RH ∼ 70%) and light illumination.

## Supporting Information Available

Experimental section and supporting notes; *J*–*V* curves, GIWAXS patterns and intensity profiles, absorption spectra and PL spectra, SEM images, calculation results of the X-ray penetration depth, plane labeling, ToF-SIMS raw data; plots of Hermans orientation factor variation, lattice structures used for DFT calculations, statistical *J–V* parameters, equivalent circuits, EQE spectra, maximum power point tracking; tables of lattice parameters, interlayer distances and lattice strain, interactions, resistance of charge transfer and transport, *J*_sc_ values, *V*_oc_ loss data, *J–V* parameters (PDF)
