# Supplementary material for: Over-18%-Efficiency Quasi-2D Ruddlesden–Popper Pb–Sn Mixed Perovskite Solar Cells by Compositional Engineering
Source: ACS Energy Lett. 2023 Jun 28;8(7):3188–95. doi: 10.1021/acsenergylett.3c00853 (PMC10353033; doi:10.1021/acsenergylett.3c00853)
Supplement: Supplementary file 1 — nz3c00853_si_001.pdf [file nz3c00853_si_001.pdf]

Supporting Information

Over-18%-Efficiency Quasi-2D Ruddlesden-Popper Pb-Sn Mixed Perovskite Solar Cells by Compositional Engineering

Zhaotong Qin, Mike Pols, Minchao Qin, Jianquan Zhang, He Yan, Shuxia Tao*, and Xinhui Lu**

Experimental Section

Materials: N, N-dimethylformamide (DMF, 99.8%, anhydrous), dimethyl sulfoxide (DMSO, 99.8%, anhydrous), tin (II) iodide (SnI_2), indene-C_{60} bisadduct (ICBA), silver (99.9%) and bathocuproine (BCP, 99%) were purchased from Sigma-Aldrich. Methylammonium iodide (MAI) was purchased from Greatcell. *Iso*-butylammonium, lead iodide (II) (PbI_2), and (6,6)-Phenyl-C_{61}-butyric acid methyl ester ($PC_{61}BM$) were purchased from Xi'an Polymer Light Technology Corp. (6,6)-Phenyl-C_{71}-butyric acid methyl ester ($PC_{71}BM$) were purchased from Luminescence Technology Corp. Poly(3,4-ethylenedioxythiophene) polystyrene sulfonate (PEDOT:PSS, Al 4083) was purchased from Ossila.

Precursor solution preparation: $BA_2MA_4(Pb_xSn_{1-x})_5I_{16}$ ($<n> = 5$, $x = 0$, 0.1, 0.3, 0.5, 0.7, 1.0) perovskite precursor solutions were prepared by first mixing *iso*-BAI, MAI and MI_2 (M = Pb or Sn) in a stoichiometric ratio of 2:4:5 in a DMF & DMSO solution in which the concentration of Pb^{2+} was 1 M and the ratio of DMF:DMSO is 960:40 (v/v). The solution vessels were put on a vortex mixer at high speed, and the solutions were well-mixed within two minutes. Then the two precursor solutions were mixed in

the volume ratios corresponding with the certain lead/tin ratios and were further stirred in the vortex mixer for another two minutes. $PC_{61}BM$ was dissolved in chloroform at a concentration of 10 mg/mL, and the optimized ETL was prepared by dissolving 1 mg $PC_{61}BM$, 1 mg $PC_{71}BM$, and 12 mg ICBA in 1 mL chloroform. Chloroform for the optimized electron transporting layer (ETL) solutions was added right before use to prevent the aggregation of the fullerene derivatives. BCP was dissolved in isopropanol (IPA) at a concentration of 0.5 mg/mL.

Device fabrication: ITO glasses were cleaned in aqueous detergent solution, deionized water, acetone, and isopropanol in sequence in a hypersonic cleaner, and then were dried and treated with UV-ozone for 30 min. The PEDOT:PSS solution was spin-coated onto the ITO substrates at 4000 rpm for 30 s and then annealed at 130°C for 30 min in air ambient. The coated substrates were transferred into a nitrogen-filled glovebox. The substrates were heated to 100°C before use. The perovskite films were spin-coated onto the substrates at 5000 rpm for 20 s and were immediately annealed at 100 °C for 10 min. Then the ETL solutions (either $PC_{61}BM$ or the optimized mixture) and BCP solutions were subsequently spin-coated both at 4000 rpm for 20 s. Finally, a 100 nm silver electrode was thermal evaporated onto the films. The effective area of the device was 4.75 mm^2, defined by a mask.

Characterization: The X-Ray Diffraction (XRD) characterization was carried out by Rigaku Smartlab using a Cu Kα X-ray source (1.54 Å). The steady-state photoluminescence (PL) was characterized with an excitation wavelength of 532 nm and the laser power of 10 mW cm^{-2} on the instrument of Horiba Raman spectrometer with photoluminescence. The UV-Vis absorption measurements were carried out using

PerkinElmer LAMBDA 950 UV/Vis/NIR Spectrophotometer. The scanning electron microscopy (SEM) images were obtained by JEOL JSM-7800F Schottky Field Emission Scanning Electron Microscope. The grazing-incidence wide-angle X-ray scattering (GIWAXS) measurements were carried out with a Xeuss 2.0 SAXS/WAXS laboratory beamline using a Cu Kα X-ray source (8.05 keV, 1.54 Å) and a Pilatus 3r 300 K detector. The focusing ion beam (FIB) process and cross-sectional SEM imaging were conducted by Thermo Scientific Scios 2 Dualbeam. The time-of-flight secondary ion mass spectroscopy (ToF-SIMS) spectra were acquired using a ToF-SIMS V spectrometer from ION-TOF GmbH. The analysis was carried out with a Bi^{3+} beam operating at 25 keV under a vacuum of ~1×10^{-9} Torr. The pulsed current of the Bi^{3+} beam was 1.0 pA. The reconstructed area was 50×50 μm^2 for all the samples. The current density-voltage (*J-V*) curves were measured by Keithley 2400 source meter under an AM 1.5G simulating source from Enlitech Solar Simulator. The specific parameters of *J-V* scan are as follows: the interval was 50 ms; the step was 0.02 V; the range was from -0.2 V to 1.2 V; no device preconditioning prior to testing was conducted; the testing condition was in a N_2-filled glovebox at room temperature (~25 °C); no antireflection coating was used; the device contact area was 4.75 mm^2 defined by mask and was calibrated by an optical microscope; the whole devices were illuminated; the reference cell was calibrated in May 2017. The external quantum efficiency (EQE) results were measured by Enlitech Photovoltaic Quantum Efficiency system. The electrochemical impedance spectroscopy (EIS) measurements were conducted using Zahner Electrochemical workstation.

Theoretical calculations Density functional theory (DFT) calculations were done to compare the different geometries and interactions in the various perovskites. The

calculations were performed with the Viennia Ab-initio Simulation Package (VASP) [1-3] using the projector augmented wave (PAW) method [4]. Electronic exchange-correlation interactions were modeled in the generalized gradient approximation (GGA) as defined by Perdew, Burke, and Ernzerhof (PBE) [5]. The outermost electrons of H ($1s^1$), C ($2s^22p^2$), N ($2s^22p^3$), Sn ($5s^25p^2$), I ($5s^25p^5$) and Pb ($6s^26p^2$) were treated as valence electrons. Dispersive interactions were accounted for through the DFT-D3 Becke-Johnson damped correction scheme as proposed by Grimme et al. [6]. The plane wave basis set was expanded to 500 eV in all calculations, where the reciprocal space was sampled with a 4 x 4 x 3 and 1 x 3 x 3 Γ-centered *k*-mesh [7] for the 3D and 2D structures, respectively. Optimized structures were obtained by allowing the ionic positions, cell shape and cell volume to change, while constraining the unit cell to an orthorhombic geometry. To analyze the strength of ionic and covalent bonds in the perovskites, net atomic charges and bond orders were obtained with the DDEC6 charge partitioning scheme for the optimized structures [8-9].

Supporting Notes

Geometry analysis: The lattice vectors of the density functional theory (DFT) optimized 3D and 2D structures of the halide perovskites (**Figure S15**) are given in **Table S5**. To probe the possibility of vertical growth of a 2D perovskite on the 3D perovskites, we calculate the strain ε of the potential interfaces as following: $\varepsilon_x = (d/2 - x) / x$, where x is any of the pseudocubic lattice vectors of the 3D perovskite and d is the distance between two consecutive layers of Pb atoms along the *a*-direction, separated by long ligands (**Table S6**). We observe that a substitution of Pb (1.6%) by Sn (2.0%) results in greater strain, resulting from the smaller size of Sn ions when compared to Pb ions.

Chemical interactions: We distinguish between three types of chemical bonding interactions in metal halide perovskites: ionic interactions, covalent interactions and hydrogen bonds. We characterize the chemical interactions using the DDEC6 atomic population analysis. [8-9] An overview of the (inter)atomic properties is given in **Table S7** and **Table S8** for 3D and 2D perovskites, respectively. Ionic interactions are characterized by the atomic charges q expressed in the elementary charge e. Covalent interactions in the framework are quantified average bond order ($\overline{\mathrm{BO}}$) between the metal species (Pb/Sn) and I anions in the inorganic framework. Hydrogen bonds are described using the total bond order ($\mathrm{TBO}^{\mathrm{N-H\cdots I}}$) between the -$NH_3$ group of the organic cations and I anions in the inorganic framework.

The overview of 2D *iso*-$(BA)_2(MA)M_2I_7$ perovskites in **Table S7** demonstrates that the inorganic framework of Pb-based perovskites is considerably more ionic (less covalent) than its Sn-based counterparts. This is reflected by: i) a larger charge on Pb (+0.69) than Sn (+0.59); ii) a larger net charge on I anions in Pb-based perovskites (-0.46) than Sn-based perovskites (-0.42); iii) a smaller overall bond order between Pb – I (0.41) than Sn – I (0.44). Interestingly, both the magnitude of the charges on *iso*-BA^+ cations and the strength of the hydrogen bonds between the cations and inorganic framework remain unaffected by the type of metal cation (M = Pb/Sn). Similar qualitative trends are found in 3D $MAMI_3$ perovskites, with minor changes to each of the type of bonds (**Table S8**). Such differences in the chemical nature of Pb and Sn play an important role in the crystallization kinetics and growth mechanisms of Pb/Sn-based perovskites and allow for the modulation of the final film quality, as is further discussed in the main text.

DFT Calculations: To better interpret the observations from experiments, we compare the geometries and the nature of the chemical bonding in Sn and Pb perovskites using DFT calculations. To focus on the major differences, we analyze two extrema compositions: $MAMI_3$ and *iso*-$(BA)_2(MA)M_2I_7$ (M = Sn/Pb). Detailed structural models and a chemical bonding analysis can be found in the Supporting Information (Note 1 and 2). Our results show that Sn-based perovskites are more covalent (less ionic) than Pb-based perovskites, regardless of their form (3D or 2D). This is reflected by an overall larger bond order between metal cations and I anions in Sn-based perovskites compare to Pb-based perovskites. We argue that the stronger covalent character of the Sn-based perovskites facilitates the rapid nucleation of the perovskite, thus resulting in the growth of a mixture of horizontal and vertical 2D phases as seen in experiments (**Figure 3e**). In contrast, Pb-based perovskite grow in a more controlled manner as a result of their smaller covalent character and the additional stabilization of the reaction intermediates that follows from the stronger electrostatic interactions between the *iso*-BA^+ cations and the Pb-based inorganic framework. We highlight this controlled growth allows for the formation of a 3D/2D interface, upon growing vertical 2D phases, that is stabilized by the covalent bonding between the 3D and 2D phases that can occur (**Figure 3h**). Moreover, we observe that the nonnegligible lattice strain that is introduced by incorporating a large cation like *iso*-BA^+ in the 3D/2D interface is smaller in Pb-rich systems (1.6%) than in Sn-rich systems (2.0%), due to the larger size of Pb compared to Sn, further supporting the experimental observations.

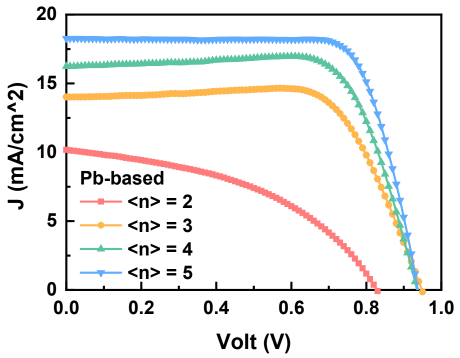

Figure S1. *J-V* curves of lead-based quasi-2D PSCs with different <*n*>.

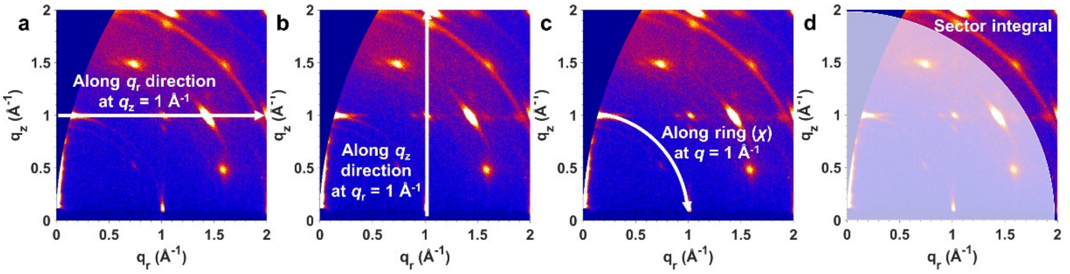

Figure S2. Schematic diagrams of GIWAXS intensity profiling (a) along q_r direction at $q_z = 1$ Å^{-1}, (a) along q_z direction at $q_r = 1$ Å^{-1}, (c) along polar angle χ at $q = 1$ Å^{-1}, and (d) of sector integral.

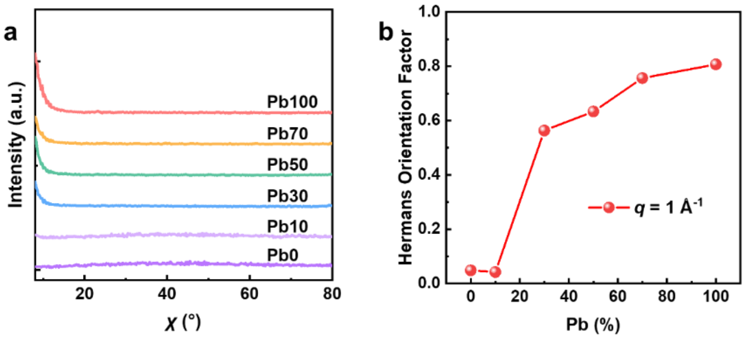

Figure S3. (a) The corresponding GIWAXS intensity profiles of **Figure 1c** along the ring at $q = 1$ Å^{-1}. (b) Hermans orientation factor of the films with various Pb/Sn ratios, calculated from the data in (a).

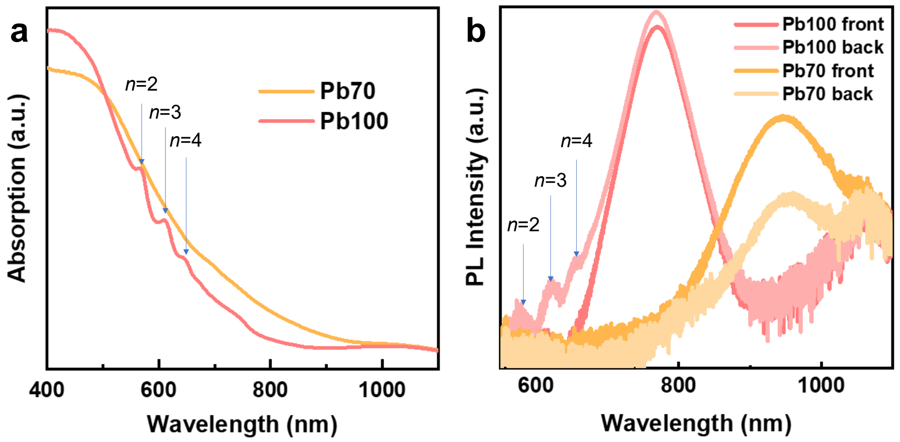

Figure S4. (a) Absorption spectra and (b) PL spectra of pure-lead and Pb70 quasi-2D perovskites with PL measured on two sides (back: near HTL and substrate; front: near ETL and silver electrode)

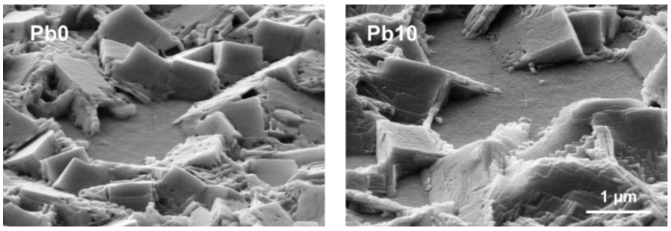

Figure S5. SEM images of 2D perovskite films with 0% and 10% lead contents.

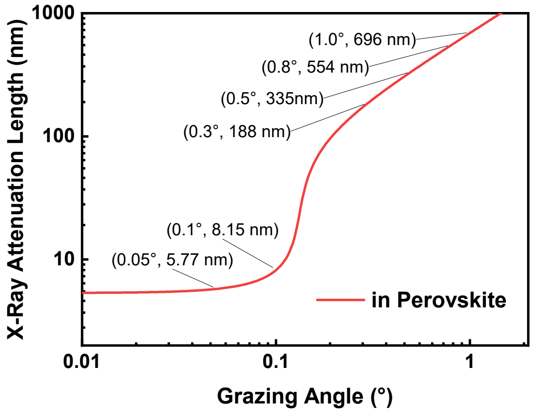

Figure S6. The calculation results of the X-ray penetration depth in the perovskite films based on previous work. The y value of each point represents the depth where the intensity of the X-ray attenuated to $1/e$ of its initial intensity at the incident angle x.[10]

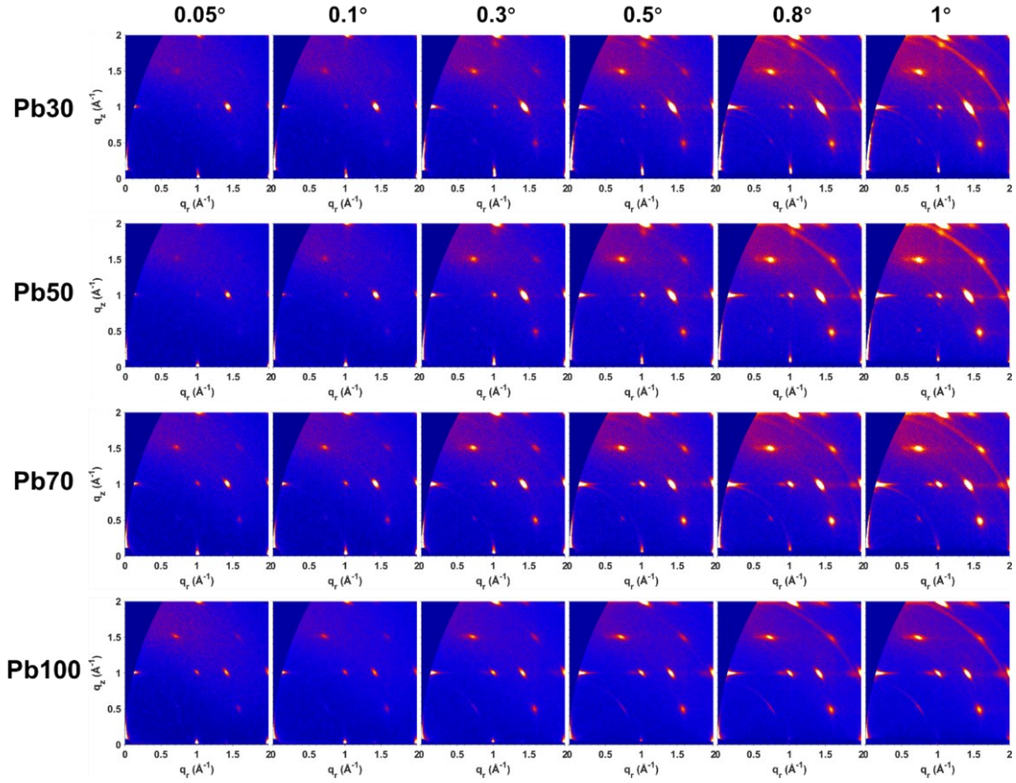

Figure S7. The complete GIWAXS patterns of the perovskite films with lead contents of 30%, 50%, 70%, and 100% at different incident angles, based on *iso*-BA$^+$.

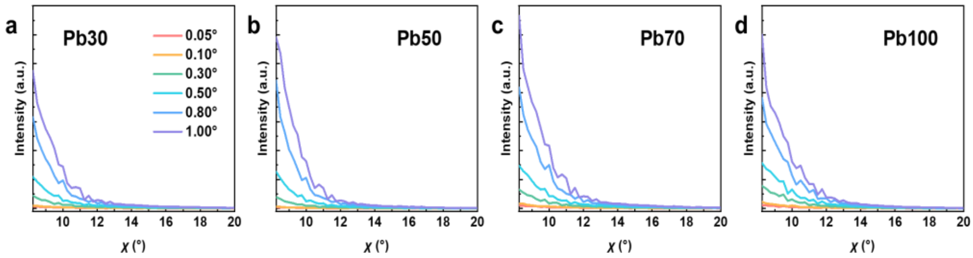

Figure S8. GIWAXS polar intensity profiles along the ring at q = 1 Å^{-1} for the 2D Pb-Sn mixed perovskite films with different Pb/Sn ratios.

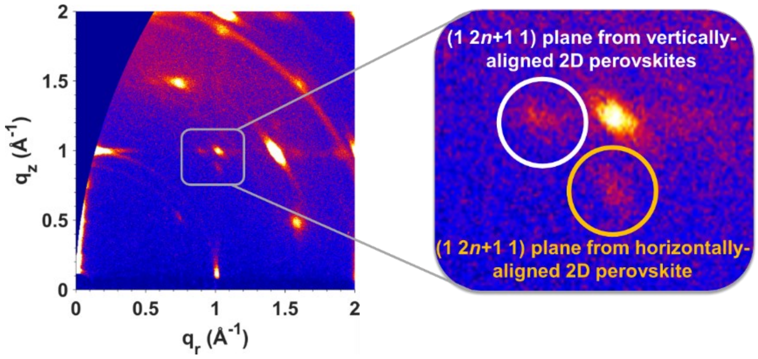

Figure S9. Labeling of the (1 2n+1 1) planes from vertically aligned and horizontally aligned 2D perovskites, where n represents the numbers of [PbI_6] octahedra along the b axis in the 2D perovskites.

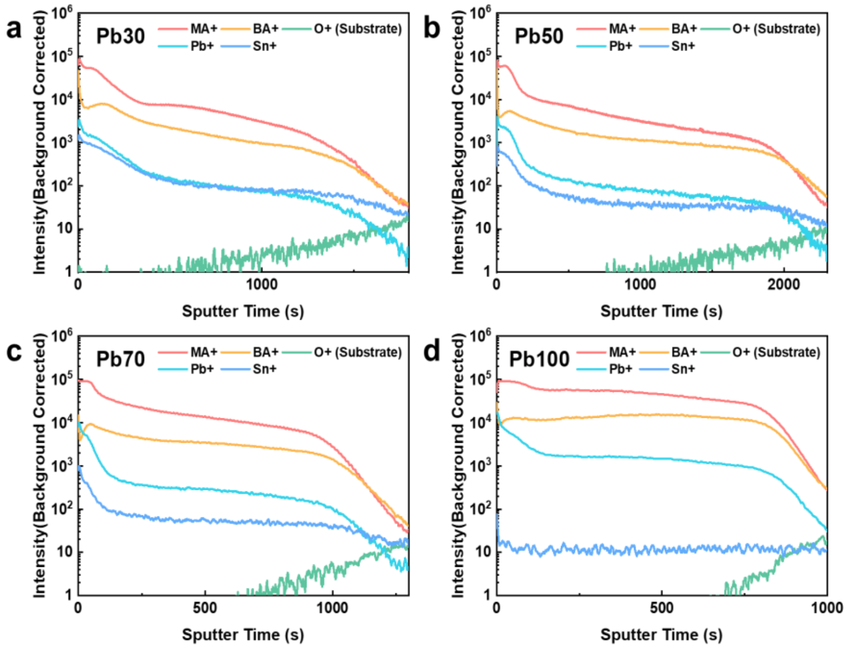

Figure S10. Raw data of ToF-SIMS measurements for the (a) Pb30, (b) Pb50, (c) Pb70, and (d) Pb100 films.

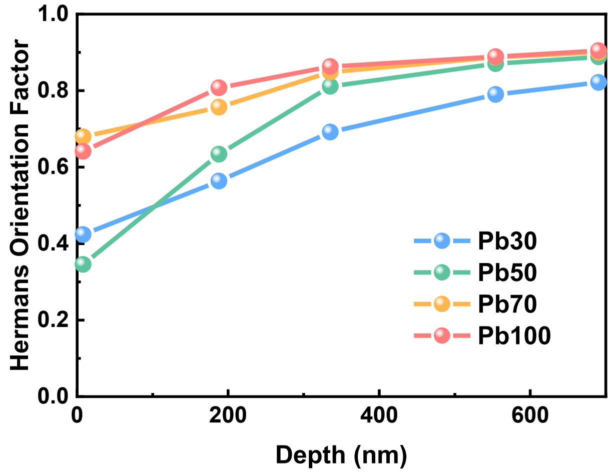

Figure S11. Plots of Hermans orientation factor variation versus the X-ray penetration depth for the Pb30, Pb50, Pb70, and Pb100 films.

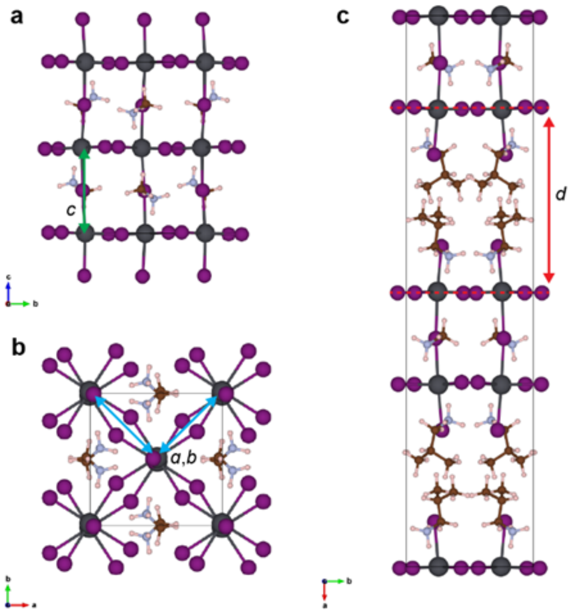

Figure S12. 3D and 2D structures of the halide perovskite structures investigated with DFT calculations. a) Top and b) side view of 3D $MAPbI_3$. c) Side view of 2D (n = 2) *iso*-$(BA)_2(MA)Pb_2I_7$ perovskite. The interlayer distances (red) and pseudocubic lattice vectors *a, b* (blue) and *c* (green) are indicated with arrows.

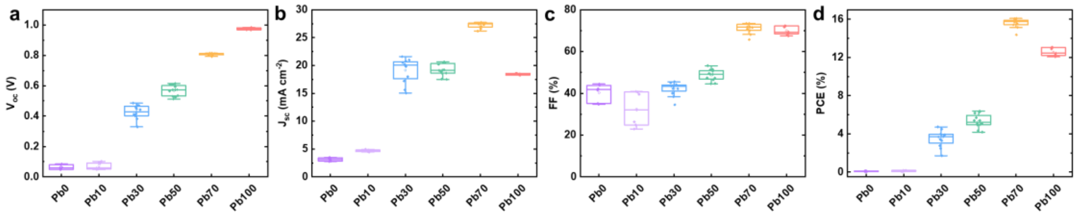

Figure S13. Statistical *J-V* results of *iso*-BA$^+$ based 2D Pb-Sn mixed PSCs from Pb0 to 100 of (a) open-circuit voltage (V_{oc}), (b) short-circuit current density (J_{sc}), (c) fill factor (FF), and (d) power conversion efficiency (PCE).

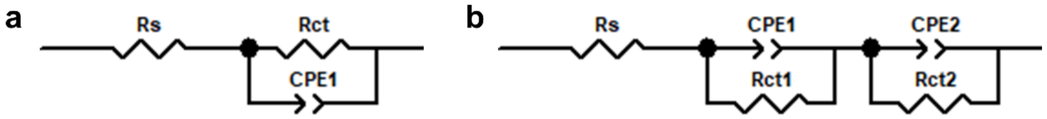

Figure S14. The equivalent circuits of (a) 1-RC model for Pb30, Pb50 and Pb70 PSCs and (b) 2-RC model for Pb100 PSCs.

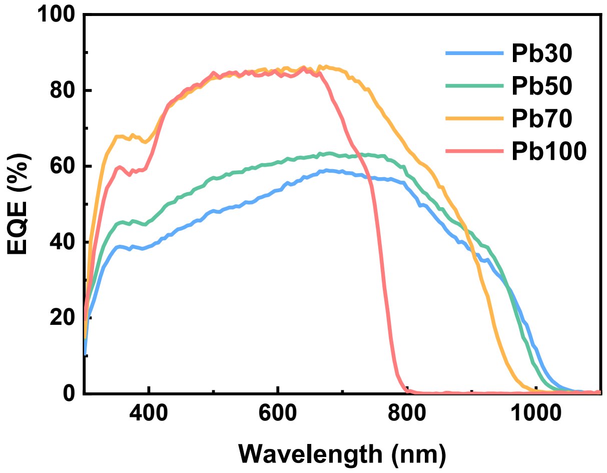

Figure S15. EQE curves of *iso*-BA$^+$ based 2D Pb-Sn mixed PSCs from Pb30 to 100.

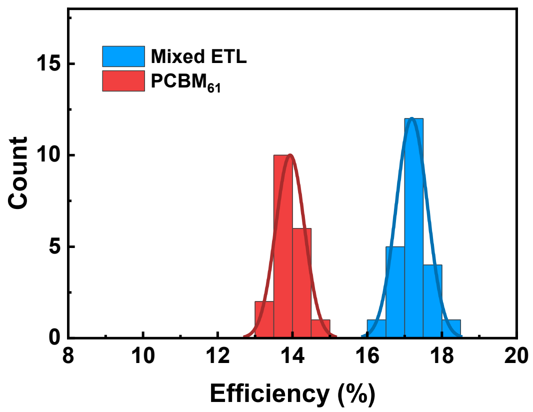

Figure S16. The histograms of the *iso*-BA^+ based Pb70 devices based on ETL of the optimized mixed ETL and $PCBM_{61}$.

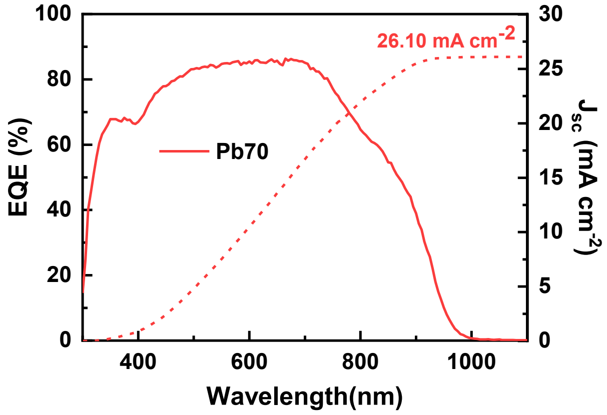

Figure S17. The EQE spectrum and the corresponding short-circuit current integral of the *iso*-BA^+ based 2D Pb70 devices.

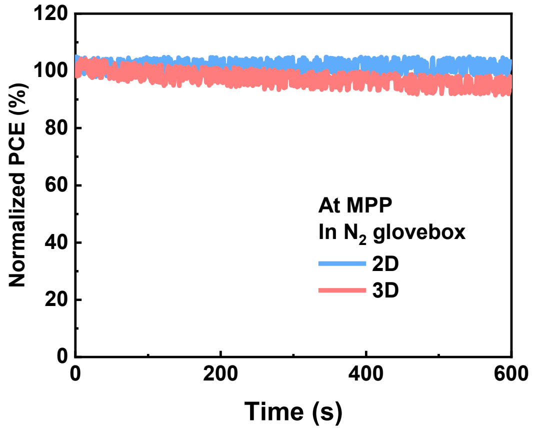

Figure S18. Maximum power point tracking of 2D and 3D Pb-Sn mixed PSCs with 70% lead content.

Table S1. An overview of the lattice parameters for the optimized 3D and 2D halide perovskites.

Phase	Perovskite	Lattice vectors			Pseudocubic lattice vectors		
		a (Å)	*b* (Å)	*c* (Å)	*a* (Å)	*b* (Å)	*c* (Å)
3D	$MAPbI_3$	8.830	8.830	12.615	6.244	6.244	6.308
	$MASnI_3$	8.739	8.739	12.402	6.179	6.179	6.201
2D	*iso*-$(BA)_2(MA)Pb_2I_7$	38.522	8.857	8.605	-	6.263	6.085
	iso-$(BA)_2(MA)Sn_2I_7$	38.06	8.786	8.61	-	6.213	6.088

Table S2. Interlayer distances *d* and space taken up by ligand *d*/2 for different ligands in a 2D perovskite. The lattice strain ε quantifies the required strain for the growth of 2D perovskites on the 3D perovskites. The reported average strain is calculated as: $\varepsilon_{avg} = (\varepsilon_a \cdot \varepsilon_b \cdot \varepsilon_c)^{1/3}$.

Perovskite	*d* (Å)	*d*/2 (Å)	$\varepsilon_{a,b}$ (%)	ε_c (%)	ε_{avg} (%)
iso-$(BA)_2(MA)Pb_2I_7$	12.734	6.367	2	0.9	1.6
iso-$(BA)_2(MA)Sn_2I_7$	12.617	6.309	2.1	1.7	2

Table S3. Different interactions between various chemical species in 2D halide perovskites with the *iso*-BA^+ ligand.

	Quantity	*iso*-$(BA)_2(MA)Pb_2I_7$	*iso*-$(BA)_2(MA)Sn_2I_7$
Ionic interactions	q_{MA}	+0.62	+0.61
	$q_{iso\text{-}BA}$	+0.60	+0.59
	q_{M}	+0.69	+0.59
	q_{I}	-0.46	-0.42
Covalent interactions	$\overline{BO}^{B-I}$	0.41	0.44
Hydrogen bonds	$TBO_{MA}^{N-H\cdots I}$	0.37	0.38
	$TBO_{iso-BA}^{N-H\cdots I}$	0.40	0.40

Table S4. Different interactions between various chemical species in 3D halide perovskites.

	Quantity	$MAPbI_3$	$MASnI_3$
Ionic interactions	q_{MA}	+0.63	+0.62
	q_{M}	+0.73	+0.62
	q_{I}	-0.45	-0.41
Covalent interactions	$\overline{BO}^{B-I}$	0.41	0.45
Hydrogen bonds	$TBO_{MA}^{N-H\cdots I}$	0.34	0.32

Table S5. The resistance of charge transfer and transport of the *iso*-BA^+ based 2D perovskite solar cells. The Pb30, Pb50 and Pb70 samples are fitted in 1-RC model, while the Pb100 sample is fitted in 2-RC model.

1-RC model	Pb30	Pb50	Pb70	Pb100
R_s/Ω	28.4	43.0	41.6	-
CPE-T/nF	6.01	7.19	13.8	-
CPE-P	0.985	0.974	0.895	-
R_{ct}/Ω	17696	14179	6595	-

2-RC model	Pb30	Pb50	Pb70	Pb100
R_s/Ω	-	-	-	102.7
CPE_1-T/nF	-	-	-	9.57
CPE_1-P	-	-	-	0.889
R_{ct1}/Ω	-	-	-	3935
CPE_2-T/nF	-	-	-	83.6
CPE_2-P	-	-	-	0.930
R_{ct2}/Ω	-	-	-	9923

Table S6. The comparison of J_{sc} values from *J-V* curves and EQE spectra.

Pb%		30	50	70	100
J_{sc} (mA cm^{-2})	From *J-V*	19.16	20.72	27.20	19.94
	From EQE	19.42	20.4	26.12	19.21
	Error (%)	-1.4	1.5	4.0	3.7

Table S7. The V_{oc} loss data of the *iso*-BA^+ based 2D perovskite solar cells.

Pb%	0	10	30	50	70	100
Maximum V_{oc} (V)	0.085	0.102	0.485	0.614	0.814	0.983
Bandgap from PL (eV)	1.289	1.269	1.258	1.254	1.311	1.612
V_{oc} loss (V)	1.204	1.167	0.773	0.640	0.497	0.629

Table S8. The *J-V* parameters of the champion device

Scan	V_{oc} (V)	J_{sc} (mA cm^{-2})	FF (%)	PCE (%)
Reverse	0.884	27.30	74.85	18.07
Forward	0.879	27.28	72.85	17.48

References

1. Kresse, G.; Hafner, J., Ab initio molecular-dynamics simulation of the liquid-metal–amorphous-semiconductor transition in germanium. *Physical Review B* **1994,** *49* (20), 14251.

2. Kresse, G.; Furthmüller, J., Efficiency of ab-initio total energy calculations for metals and semiconductors using a plane-wave basis set. *Computational materials science* **1996,** *6* (1), 15-50.

3. Kresse, G.; Furthmüller, J., Efficient iterative schemes for ab initio total-energy calculations using a plane-wave basis set. *Physical review B* **1996,** *54* (16), 11169.

4. Kresse, G.; Joubert, D., From ultrasoft pseudopotentials to the projector augmented-wave method. *Physical review b* **1999,** *59* (3), 1758.

5. Perdew, J. P.; Burke, K.; Ernzerhof, M., Generalized gradient approximation made simple. *Physical review letters* **1996,** *77* (18), 3865.

6. Grimme, S.; Ehrlich, S.; Goerigk, L., Effect of the damping function in dispersion corrected density functional theory. *Journal of computational chemistry* **2011,** *32* (7), 1456-1465.

7. Monkhorst, H. J.; Pack, J. D., Special points for Brillouin-zone integrations. *Physical review B* **1976,** *13* (12), 5188.

8. Manz, T. A.; Limas, N. G., Introducing DDEC6 atomic population analysis: part 1. Charge partitioning theory and methodology. *RSC advances* **2016,** *6* (53), 47771-47801.

9. Manz, T. A., Introducing DDEC6 atomic population analysis: part 3. Comprehensive method to compute bond orders. *RSC advances* **2017,** *7* (72), 45552-45581.

10. Qin, M.; Xue, H.; Zhang, H.; Hu, H.; Liu, K.; Li, Y.; Qin, Z.; Ma, J.; Zhu, H.; Yan, K.; Fang, G.; Li, G.; Jeng, U. S.; Brocks, G.; Tao, S.; Lu, X., Precise Control of Perovskite Crystallization Kinetics via Sequential A-Site Doping. *Advanced Materials* **2020,** *32* (42), 2004630.
